# Association of tirofiban treatment with outcomes following endovascular therapy in cardioembolic stroke: insights from the RESCUE BT randomized trial

**DOI:** 10.1186/s40001-023-01406-x

**Published:** 2023-11-01

**Authors:** Benbing Rong, Zhangbao Guo, Lijie Gao, Yuan Yang, Wenjie Zi, Zhongming Qiu, Fengli Li, Zhiyu Lv, Ying luo, Renliang Meng, Yang Xie, Ting Long, Shujiang Zhang, Jinshan Jiang, Jinfeng Tian, Jingling Zhao, Hongliang Zeng, Zhengzhou Yuan

**Affiliations:** 1 Department of Neurology, Affiliated Hospital of Southwest Medical University, Luzhou, China; 2 Department of Neurology, Wuhan No. 1 Hospital, Wuhan, China; 3 Department of Rehabilitation, Zhujiang Hospital, Southern Medical University, Guangzhou, China; 4 Department of Neurology, Xinqiao Hospital and The Second Affiliated Hospital, Army Medical University (Third Military Medical University), Chongqing, China; 5 Department of Neurology, The 903Rd Hospital of The Chinese People’s Liberation Army, Hangzhou, China; 6 School of Clinical Medicine, Southwest Medical University, Luzhou, China; 7 Department of Neurology, Ganzhou People’s Hospital, Zhanggong District, 17 Hongqi Avenue, Ganzhou, China

**Keywords:** Acute ischemic Stroke, Tirofiban, Endovascular therapy, Cardio-embolism

## Abstract

**Background and purpose:**

The efficacy and safety of tirofiban in endovascular therapy for cardioembolic ischemic stroke patients remain controversial. This study aimed to evaluate the role of intravenous tirofiban before endovascular therapy in cardioembolic stroke.

**Methods:**

This post hoc analysis utilized data from the RESCUE BT (Endovascular Treatment With versus Without Tirofiban for Patients with Large Vessel Occlusion Stroke) trial, which was an investigator-initiated, randomized, double-blind, placebo-controlled trial. Participants were randomized to receive either tirofiban or a placebo in a 1:1 ratio before undergoing endovascular therapy. The study included patients aged 18 years or older, presenting with occlusion of the internal carotid artery or middle cerebral artery (MCA) M1/M2 within 24 h of the last known well time, and with a stroke etiology of cardioembolism. The primary efficacy outcome was global disability at 90 days, assessed using the modified Rankin Scale (mRS). The safety outcome included symptomatic intracranial hemorrhage (sICH) within 48 h and mortality within 90 days.

**Results:**

A total of 406 cardioembolic stroke patients were included in this study, with 212 assigned to the tirofiban group and 194 assigned to the placebo group. Tirofiban treatment did not correlate with a favorable shift towards a lower 90-day mRS score (adjusted common odds ratio [OR], 0.91; 95% CI 0.64–1.3; *p* = 0.617). However, the tirofiban group had a significantly higher risk of symptomatic intracranial hemorrhage (sICH) within 48 h (adjusted OR, 3.26; 95% CI 1.4–7.57; *p* = 0.006) compared to the placebo group. The adjusted odds ratio (aOR) for mortality within 90 days was 1.48 (95% CI 0.88–2.52; *p* = 0.143).

**Conclusions:**

Tirofiban treatment was not associated with a lower level of disability and increased the incidence of sICH after endovascular therapy in cardioembolic stroke patients.

## Introduction

Ischemic stroke remains a significant global health burden, contributing to substantial morbidity and mortality rates [16]. Endovascular therapy (EVT) has revolutionized the management of acute ischemic stroke caused by large vessel occlusion (LVO), offering improved outcomes compared to conventional medical therapy alone [5]. While the efficacy of EVT is well-established, there is a need to further refine treatment strategies and optimize patient outcomes [10]. Cardiac embolism represents a common etiology of LVO-induced ischemic stroke, accounting for a significant proportion of cases undergoing EVT [6]. The embolization of cardiac-origin thrombi or atheromatous debris from the heart's chambers or major vessels can lead to the occlusion of intracranial arteries, resulting in devastating neurological deficits. Despite advancements in endovascular techniques, the management of cardioembolic stroke remains challenging.

Tirofiban, a glycoprotein IIb/IIIa receptor inhibitor, has gained attention as a potential adjunctive therapy in the context of EVT for cardioembolic stroke [24]. By inhibiting platelet aggregation and preventing arterial thrombosis, tirofiban holds promise in enhancing recanalization rates and improving clinical outcomes [17]. However, there are conflicting findings regarding the efficacy and safety of tirofiban in this specific patient population, and previous studies have not exclusively focused on the cardio-embolism subgroup [8, 23]. The RESCUE BT (Endovascular Treatment With versus Without Tirofiban for Patients with Large Vessel Occlusion Stroke) trial sought to evaluate the impact of tirofiban in combination with EVT for patients with proximal intracranial LVO [15]. Although the trial did not demonstrate a significant improvement in functional disability at 90 days with the administration of intravenous tirofiban compared to placebo, the question of whether tirofiban could offer benefits to patients with cardioembolic stroke remains unclear.

In this secondary analysis of the RESCUE BT trial [15], we aimed to investigate the association between intravenous tirofiban and clinical outcomes after EVT in patients specifically presenting with LVO attributable to cardio-embolism.

## Methods

### Study design and participants

The RESCUE BT trial was a prospective, investigator-initiated, double-blind, randomized, placebo-controlled trial aimed at assessing the safety and efficacy of intravenous tirofiban versus placebo prior to EVT in acute ischemic stroke with large vessel occlusion from 55 hospitals in China between October 2018 and October 2021. The trial protocol and primary results had been published [14, 15]. The RESCUE BT trial was prospectively registered on the Chinese Clinical Trial Registry (chictr.org.cn, ChiCTR-INR-17014167) and adhered to the principles outlined in the Declaration of Helsinki. The study protocol received approval from the ethics committee of Xinqiao Hospital, Army Medical University, as well as the relevant ethics committees at all participating centers. Prior to enrollment in the trial, written informed consent was obtained from all patients or their authorized representatives, ensuring their voluntary participation and understanding of the study’s objectives, procedures, and potential risks.

Patients who were aged 18 years old or more, presenting with occlusion of the internal carotid artery (ICA) or middle cerebral artery (MCA) -M1/M2 within 24 h of time last known well, and with a stroke etiology of cardio-embolism were included in the present study. For patients who experienced a stroke within 48 h of Direct Oral Anticoagulant (DOAC) intake, they did not receive thrombolytic treatment. In patients with a history of warfarin anticoagulation therapy, intravenous thrombolysis was considered if INR ≤ 1.7 or a PT < 15 s, with the informed consent of patients or their family members.

### Etiological identification of Cardiac embolism

In this study, we implemented a meticulous and comprehensive approach for the etiological identification of cardioembolic stroke. Data were collected from various sources, including case report forms and source documents, encompassing comprehensive medical history, clinical features, laboratory tests, 24-h electrocardiograms, echocardiography, non-invasive brain imaging, and DICOM format angiography. Two experienced neurologists, blinded to treatment allocation, centrally assessed the data. The criteria for cardioembolic-related LVO were optimized based on the Trial of Org 10172 in Acute Stroke Treatment [1], with additional considerations for excluding other potential etiologies and supporting evidence of embolic occlusion on angiography. Consensus was achieved through discussions in cases of discrepancies. The diagnosis of cardioembolic stroke relies on the integration of clinical, neuroimaging, and cardiac and vascular evaluations. Clinical presentation includes a sudden onset of maximal neurological deficits. Neuroimaging reveals cortical lesions in a cerebral territory and may demonstrate involvement of multiple arterial territories. Vascular evaluation excludes large-artery plaque, while cardiac evaluation identifies high-risk cardiac conditions, such as atrial fibrillation, patent foramen ovale, or endocarditis [3, 7]. The presence of a typical clinical presentation, neuroimaging findings, positive evidence of a high-risk cardiac source, and exclusion of large-artery plaque collectively establish the diagnosis of cardioembolic stroke.

### Treatments

Eligible subjects were randomly assigned to the tirofiban group or the control group. The tirofiban group received intravenous bolus and continuous infusion of tirofiban, while the control group received saline placebo. Tirofiban was administered as a 10 μg/kg bolus, followed by a maintenance infusion of 0.15 μg/kg/min for up to 24 h. The specific endovascular treatment techniques, such as stent-retrieval, local aspiration, angioplasty, and stenting, were performed based on the neurointerventionalists' discretion. However, the use of intra-arterial thrombolytics (e.g., alteplase or urokinase), tirofiban, rescue drug, or other glycoprotein IIb/IIIa inhibitors was not recommended. The rescue drug, available in the study kits, could be used if antegrade blood flow could not be maintained after angioplasty and/or stenting, following the same administration protocol as the study drug. Patients experiencing deteriorating symptoms underwent an immediate repeat of the routine or dual-source head CT scan. For those patients whose condition remained stable, a routine or dual-source head CT scan was conducted 20 h post-procedure. At the 20th hour, all patients without contraindications received oral antiplatelet therapy with aspirin and/or clopidogrel tablets. Patients undergoing angioplasty/stenting received dual-antiplatelet therapy with aspirin and clopidogrel, while others received mono-antiplatelet therapy. The study drug was discontinued at the 24th hour, and subsequent patient management followed the current stroke management guidelines. Intravenous heparin could be used during the thrombectomy procedure, as determined by the operator. Additionally, subcutaneous heparin or low-molecular-weight heparin could be administered post-procedure for deep vein thrombosis prophylaxis. However, the use of other anticoagulants or antiplatelet agents was prohibited within the first 24 h post-randomization.

### Variables and imaging assessment

During the enrollment process, demographic variables, vascular risk factors, baseline NIHSS score, treatment information, and workflow measures were recorded. To ensure consistency and reliability in the evaluation process, all imaging data were reviewed by the imaging core laboratory of the RESCUE BT. The determination of the occlusion site was made based on the findings from CT or MR angiography conducted upon admission, with specific classification into distinct categories including internal carotid artery (ICA), middle cerebral artery M1 segment (M1 MCA), or middle cerebral artery M2 segment (M2 MCA). The extent of ischemic injury was quantified by employing the widely recognized Alberta Stroke Program Early CT Score (ASPECTS). The evaluation of reperfusion status during the final angiography utilized the expanded Thrombolysis in Cerebral Ischemia (eTICI) score [9], which encompasses various grades (2b, 2c, and 3) that correspond to different degrees of reperfusion, ranging from substantial to near-complete or complete reestablishment of blood flow in the affected area.

### Outcomes

The primary outcome of this study was to assess the proportion of patients achieving functional independence after 90 days, defined as a modified Rankin Scale (mRS) score of 0–2 [18]. To ensure consistency, the neurologists thoroughly reviewed structured video or voice recordings of the patients. There were no dropouts during the follow-up period. The key secondary outcome was disability level measured by the mRS scores at 90 days. Safety outcomes included the occurrence of all-cause death within 90 days, any intracranial hemorrhage (ICH), and symptomatic intracranial hemorrhage (sICH) within 48 h. sICH was assessed according to the Heidelberg criteria [21], while symptomatic hemorrhage was defined as the presence of ICH accompanied by a clinical deterioration of 4 or more points on the National Institutes of Health Stroke Scale (NIHSS) score [4].

### Statistical analysis

Patient characteristics and clinical outcomes in the two groups were reported using descriptive statistics, including number (percentage) or median and interquartile range. Categorical variables were analyzed using the χ [2] or Fisher exact tests, while continuous variables were assessed using the Student *t* and Mann–Whitney *U* tests. Association between tirofiban treatment and outcomes were analyzed using multivariable binary logistic regression, ordinal logistic regression, and linear regression model as appropriate, adjusting for age, baseline NIHSS score and APSECTS, onset to randomization time, and location of occlusion. Unadjusted and adjusted common odds ratio (OR), odds ratio, and beta coefficient were reported with corresponding 95% confidence intervals (CIs) to indicate statistical precision. No imputation was performed for there were no missing values of variables included in the multivariable regression models. No patient lost follow-up. All statistical analyses were based on the intention-to-treat population with the per-protocol population as sensitivity analyses. Patients who actually received the allocated treatment and did not have major protocol violations were included in the per-protocol analysis. All tests were two-sided, and statistical significance was set at a *P*-value of < 0.05. Statistical analyses were performed using SPSS (version 23.0, IBM). Figures were drawn using STATA version 15.2 (StataCorp LLC, TX, USA).

## Results

### Patient characteristics

Among the 950 patients initially randomized in the RESCUE BT trial, after excluding 2 patients who withdrew informed consent, 425 patients with intracranial atherosclerotic disease (ICAD), 77 patients with unknown stroke etiology, and 30 patients with other stroke etiology, a total of 406 patients with acute ischemic stroke due to cardio-embolism were included in this analysis. Of the included patients, 212 were assigned to the tirofiban group and 194 patients were assigned to the placebo group.

The demographic characteristics of the patient cohort were as follows: the median age was 69 years (interquartile range, IQR: 59–75), and the median NIHSS score was 16 (IQR: 12–19). The male-to-female ratio among the included patients was 44.1%. Table 1 provides a comprehensive overview of the baseline characteristics and treatment details for both arms of the study. The baseline characteristics, including age, baseline NIHSS score, ASPECTS, and onset to recanalization were well-balanced between the two groups. However, the median time from onset to randomization was significantly shorter in the placebo group compared to the tirofiban group (313(203–483) *vs.* 350(236–527), *p* = 0.045) (Table 1).

**Table 1:** baseline characteristics of participants

	Total (*N* = 406)	Placebo (*n* = 194)	Tirofiban (*n* = 212)	*P* value
Age, y	69 (59–75)	68(57–76)	69(59–78)	0.442
Male, *n* (%)	179(44.1)	86(44.3)	93(43.9)	0.079
Smoking, *n* (%)	47(11.6)	19(9.8)	28(13.2)	0.07
Medical history, *n* (%)				
Coronary heart disease	74(18.2)	40(20.6)	34(16)	0.05
Atrial fibrillation	261(64.3)	124(63.9)	137(64.6)	0.082
Hypertension	197(48.5)	92(47.4)	105(49.5)	0.072
Hyperlipidemia	39(9.6)	15(7.7)	24(11.3)	0.064
Diabetes	68(16.7)	29(14.9)	39(18.4)	0.069
Ischemic stroke	85(20.9)	44(22.7)	41(19.3)	0.069
Prestroke antithrombolic history, n (%)				
History antiplatelet	41(10.1)	19(9.8)	22(10.4)	0.128
History anticoagulation	63(15.5)	31(16.0)	32(15.1)	0.106
Baseline SBP, mmHg	148 (132–166)	147 (131–164)	150 (133–169)	0.433
Serum glucose	7.0 (5.6–9.2)	7.0 (5.5–9.1)	7.0 (5.6–9.3)	0.9
Occlusion site, *n* (%)				0.061
ICA intracranial	101(24.9)	49(25.3)	52(24.5)	
MCA-M1	239(58.9)	114(58.8)	125(59)	
MCA-M2	66(16.3)	31(16)	35(16.5)	
Substantial reperfusion, *n* (%)	386(95.1)	183(94.3)	203(95.8)	0.146
Anesthesia, *n* (%)				0.087
General	105(25.9)	49(25.3)	56(26.4)	
Local	301(74.1)	145(74.7)	156(73.6)	
Baseline NIHSS	16 (12–19)	15 (11–18)	16 (12–20)	0.076
Baseline ASPECTS	8 (7–9)	8 (7–9)	8 (7–9)	0.456
Onset to randomization, min	334(223–505)	313(203–483)	350(236–527)	**0.045**
Onset to recanalization, min	389(280–577)	359(268–546)	405(289–587)	0.054
Total passes	2 (2–3)	2 (1–3)	2 (2–3)	0.051
ASITN-SIR	2 (2–3)	2 (2–3)	2 (2–3)	0.508

Values are presented as number (%), mean ± standard deviation, or median (interquartile range)

*SBP* systolic blood pressure, *ICA* internal carotid artery, *MCA* middle cerebral artery, *NIHSS* National Institutes of Health Stroke Scale, *ASPECTS* Alberta Stroke Program Early CT Score, *ASITN-SIR* American Society of Interventional and Therapeutic Neuroradiology and Society of Interventional Radiology

### Efficacy outcomes

**Fig. 1:**
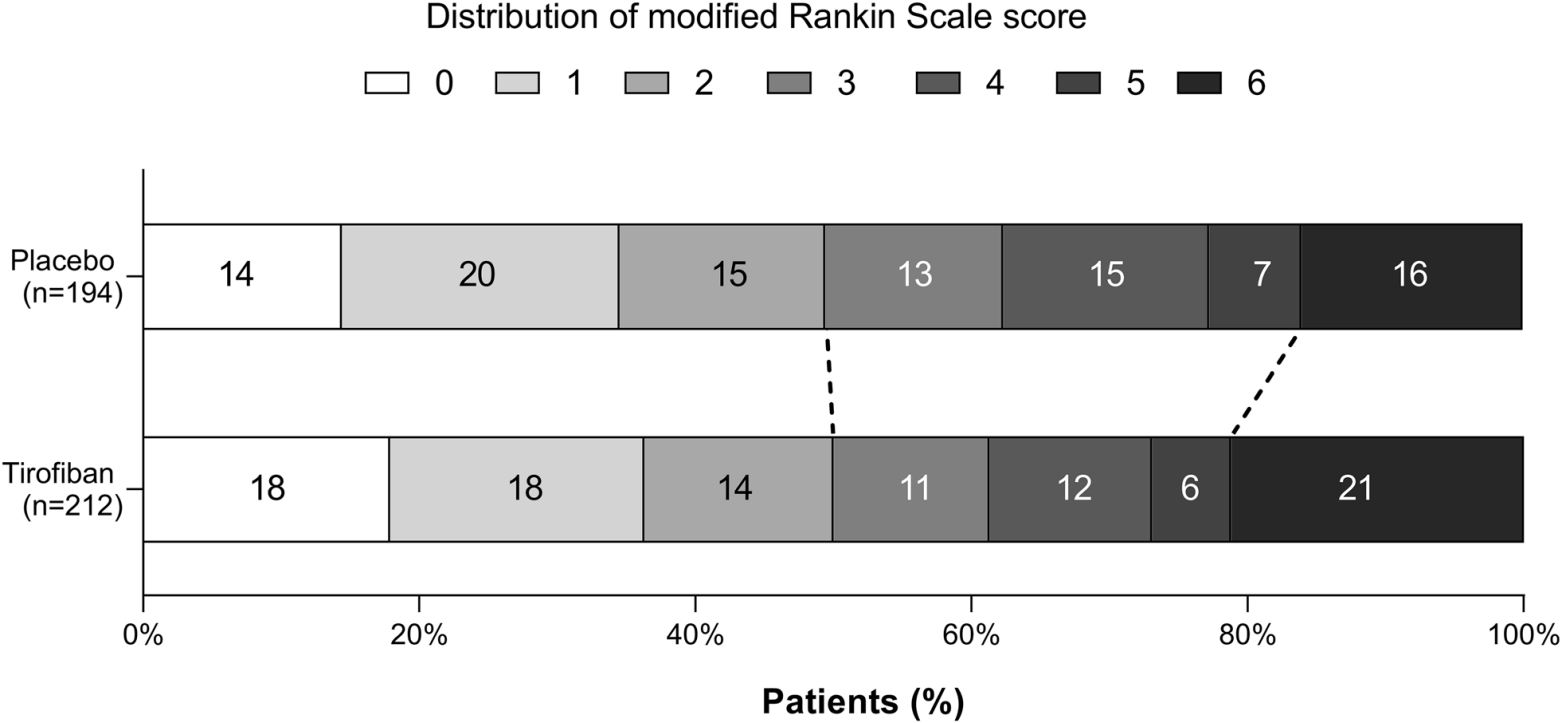
Distribution of modified Rankin Scale score

The intention-to-treat analysis comparing the two treatment arms yielded the following results, as presented in Fig. 1 and Table 2. At the 3-month follow-up, there were no statistically significant differences in the mean modified Rankin Scale (mRS) scores between the placebo and tirofiban groups (3(1–4) *vs.* 3(1–4), *p* = 0.941). Likewise, there were no significant differences in the proportions of patients achieving favorable functional outcomes, defined as mRS scores of 0–1 (35.6% vs. 37.7%, *p* = 0.681) or 0–2 (49.5% vs. 50.0%, *p* = 0.921), between the two groups. Additionally, the proportions of patients attaining an mRS score of 0–3 were comparable between the placebo and tirofiban groups (62.4% vs. 61.3%, *p* = 0.839). No significant differences were found between the placebo and tirofiban groups regarding reperfusion at 48 h, the utility of rescue drugs, changes in NIHSS scores from baseline at 24 h and 7 days, or the 3-month EuroQol-5 Dimensions 5 Levels (EQ5D5L) score.

**Table 2:** Outcomes between two treatment arms in the intention-to-treat population

	Total (*N* = 406)	Placebo (*n* = 194)	Tirofiban (*n* = 212)	*P*-value
Efficacy outcomes				
3-month mRS score	3(1–4)	3(1–4)	3(1–4)	0.941
3-month mRS score 0–1, *n* (%)	149(36.7)	69(35.6)	80(37.7)	0.681
3-month mRS score 0–2, *n* (%)	202(49.8)	96(49.5)	106(50)	0.921
3-month mRS score 0–3, *n* (%)	251(61.8)	121(62.4)	130(61.3)	0.839
NIHSS 24 h minus baseline	− 2(− 6 to 2)	− 2(− 6 to 2)	− 2(− 6 to 2)	0.825
NIHSS 7d minus baseline	− 5(− 10 to 1)	− 5(− 10 to 1)	− 5(− 10 to 2)	0.358
Reperfusion at 48 h, *n* (%)	295(96.4)	144(97.3)	151(95.6)	0.417
3-month EQ5D5L	0.7(0.2–1.0)	0.7(0.2–0.9)	0.7(0.2–1.0)	0.863
Safety outcomes				
sICH, *n* (%)	33(8.1)	7(3.6)	26(12.3)	**0.002**
Any ICH, *n* (%)	148(36.5)	62(32.1)	86(40.6)	0.078
3-month death, *n* (%)	76(18.7)	31(16)	45(21.2)	0.176

Values are presented as number (%), or median (interquartile range)

*mRS* modified Rankin Scale, *EQ-5D-5L* EuroQol 5-Dimension 5-Level, *sICH* symptomatic intracranial hemorrhage

### Safety outcomes

The incidence of symptomatic intracranial hemorrhage (sICH) was significantly higher in the tirofiban group compared to the placebo group (12.3% *vs.* 3.6%, *p* = 0.002). However, there was no significant difference observed in the incidence of any radiologic intracranial hemorrhage between the two groups (40.6% *vs.* 32.1%, *p* = 0.078). The 3-month mortality rate was 21.2% in the tirofiban group and 16.0% in the placebo group (*p* = 0.176). Further details on safety outcomes can be found in Table 2.

The association between tirofiban treatment and outcomes was assessed using both univariable and multivariable regression models, as shown in Table 3. In the univariable regression model, there were no statistically significant association between intravenous tirofiban and the 3-month modified Rankin Scale (mRS) score (common odds ratio: 0.99, 95% CI 0.7 to 1.39, *p* = 0.941). Similar results were observed after adjusting for potential confounders (adjusted common odds ratio: 0.91, 95% CI 0.64 to 1.3, *p* = 0.617). Similar results were found for the proportions of patients with an mRS score 0–1, 0–2, or 0–3, with no significant associations observed in either the unadjusted or adjusted models. Furthermore, there were no significant associations between Tirofiban and 3-month mortality in either the unadjusted (coefficient: 1.42, 95% CI 0.85 to 2.35, *p* = 0.177) or adjusted model (coefficient: 1.48, 95% CI 0.88 to 2.52, *p* = 0.143). Likewise, the occurrence of any intracranial hemorrhage (ICH) showed no significant association with Tirofiban in the unadjusted (coefficient: 1.44, 95% CI 0.96 to 2.17, *p* = 0.079) or adjusted model (coefficient: 1.44, 95% CI 0.94 to 2.19, *p* = 0.094). No significant associations were found between Tirofiban and reperfusion at 48 h, changes in NIH Stroke Scale (NIHSS) scores from baseline at 24 h and 7 days, or the 3-month EuroQol-5 Dimensions 5 Levels (EQ5D5L) score in either the unadjusted or adjusted models. However, there was a significant association between Tirofiban and hemorrhagic transformation sICH in both the unadjusted (coefficient: 3.71, 95% CI 1.57 to 8.77, *p* = 0.003) and adjusted model (coefficient: 3.85, 95% CI 1.59 to 9.09, *p* = 0.003).

**Table 3:** Association between Tirofiban and Placebo group

	Unadjusted model		Adjusted model	
	Coefficients (95% CI)	*P* value	Coefficients (95% CI)	*P* value
3-month mRS score	0.99(0.7,1.39)	0.941	0.91(0.64,1.3)	0.617
3-month mRS score 0–1	1.1(0.73,1.65)	0.651	1(0.63,1.57)	0.994
3-month mRS score 0–2	1.02(0.69,1.51)	0.917	0.97(0.63,1.51)	0.907
3-month mRS score 0–3	0.96(0.64,1.43)	0.828	0.93(0.59,1.47)	0.763
NIHSS 24 h minus baseline	0.3(− 1.49,2.08)	0.745	1.38(0.54,3.49)	0.5
NIHSS 7d minus baseline	1.29(− 1,3.57)	0.27	1.77(0.15,20.34)	0.647
Reperfusion at 48 h	1.36(0.55,3.35)	0.509	1.04(− 1.18,3.25)	0.837
3-month EQ5D5L	− 0.01(− 0.08,0.07)	0.879	0.89(− 1.56,1.91)	0.847
sICH	3.71(1.57,8.77)	0.003	3.85(1.59,9.09)	0.003
Any ICH	1.44(0.96,2.17)	0.079	1.44(0.94,2.19)	0.094
3-month death	1.42(0.85,2.35)	0.177	1.48(0.88,2.52)	0.143

Age, occlusion site, onset to randomization time, Baseline NIHSS, and baseline ASPECTS were adjusted in adjusted model

*mRS* modified Rankin Scale, *EQ-5D-5L* EuroQol 5-Dimension 5-Level, *sICH* symptomatic intracranial hemorrhage

## Discussion

This paper reports on a post hoc analysis of the RESCUE BT randomized trial, which aimed to investigate the efficacy of tirofiban for patients presenting with cardioembolic LVO within 24 h of onset. The results demonstrated that the administration of intravenous tirofiban prior to EVT didn’t improve the rate of functional independence at 90 days. However, the use of tirofiban in cardioembolic stroke patients undergoing endovascular therapy was associated with an increased risk of sICH compared to placebo. These findings suggest that tirofiban may be a harmful adjunct to endovascular therapy for patients with cardioembolic LVO.

The occurrence of sICH associated with antithrombotic drugs in endovascular therapy is a well-known concern [20]. Our findings are consistent with a previous study by Kellert et al. which reported that the use of tirofiban after thrombectomy significantly increases the risk of fatal bleeding [8]. The findings of our study also align with the MR CLEAN-MED study [19], providing evidence of increased symptomatic intracranial hemorrhage associated with tirofiban administration in AIS patients undergoing endovascular therapy. We excluded patients who received intravenous thrombolysis (IVT) or dual antiplatelet therapy, while in the MR CLEAN-MED study, the majority of patients included received intravenous thrombolysis along with the administration of antithrombotic agents. This consistency strengthens the notion that tirofiban, may contribute to a higher risk of bleeding complications in the context of endovascular stroke treatment. These results underscore the need to optimize the risk–benefit profile of tirofiban in stroke interventions, aiming to minimize bleeding while effectively preventing thrombotic events. To strike a balance between preventing thrombotic events and minimizing bleeding complications. This approach would enable the identification of patients who are more likely to experience bleeding complications and facilitate the implementation of appropriate risk mitigation strategies. However, studies by X. Pan et al. and Y. Wang et al. provide conflicting results regarding the safety of tirofiban in endovascular stroke treatment [12, 22]. X. Pan et al. evaluated the safety and efficacy of tirofiban as adjunctive therapy in patients with acute vertebrobasilar artery occlusion undergoing mechanical thrombectomy. Their findings did not show a statistically significant increase in the risk of symptomatic intracranial hemorrhage or adverse outcomes associated with tirofiban use. Similarly, Y. Wang et al. conducted a study on the overall harmful effects of aspirin and unfractionated heparin during endovascular stroke treatment in patients with successful reperfusion. Their investigation did not find notable differences in the harmful effects of these medications based on reperfusion status. Previous report subgroup of RESCUE BT shows that tirofiban is safe and can improve the good outcome in patients with ICAD related LVO [17]. However, it is important to note that these studies may not have specifically focused on cardioembolic stroke patients or conducted meticulous subgroup analyses based on stroke etiology.

The increased risk of sICH associated with tirofiban administration in cardioembolic stroke patients undergoing endovascular therapy can be attributed to several factors. Firstly, tirofiban's mechanism of action involves inhibiting platelet aggregation, which can lead to prolonged bleeding time and impaired hemostasis [25]. This antiplatelet effect is particularly concerning in patients with pre-existing vascular abnormalities or compromised blood–brain barrier integrity, commonly observed in cardioembolic stroke patients. Secondly, endovascular therapy itself carries inherent risks of vessel injury and subsequent bleeding. Tirofiban's antiplatelet effect may further exacerbate these risks by preventing the formation of platelet plugs at the site of vessel injury, thereby impairing the natural hemostatic response [11]. The combination of endovascular therapy and tirofiban administration may create a synergistic effect, amplifying the likelihood of sICH. Furthermore, it is worth noting that the dosage regimen of tirofiban utilized in this study was based on previous studies conducted in the context of myocardial infarction. This approach may have potentially contributed to a slightly elevated risk of any ICH. To establish the ideal dosage for patients with ischemic stroke, additional investigations exploring different dosage levels are warranted, considering the dose-dependent impact of tirofiban on bleeding complications.

This study possesses several limitations that should be considered when interpreting the findings. Firstly, the analysis was conducted as a post hoc analysis within the RESCUE BT trial, which may introduce inherent limitations and potential biases. This study might have been underpowered to detect a significant treatment effect within the specific subgroup of patients with cardioembolic stroke. Larger-scale randomized controlled trials specifically focusing on this patient population are essential to further validate our findings. Secondly, the tirofiban group exhibited a significantly longer time from symptom onset to randomization, leading to a delayed time to reperfusion, although the difference in the time from symptom onset to reperfusion did not reach statistical significance. Previous studies have shown that a prolonged time from symptom onset to reperfusion in patients undergoing endovascular therapy within 6 h is associated with unfavorable outcomes [2, 13]. Although our study demonstrated similar favorable outcomes in both groups and included patients with acute ischemic stroke within 24 h of symptom onset, the difference in baseline characteristic of time to randomization might have influenced the higher incidence of intracranial hemorrhage in the tirofiban group. Thirdly, the inclusion of patients with Patent Foramen Ovale (PFO) in the cardioembolic stroke group without assessing the RoPe (Risk of Paradoxical Embolism) score represents a significant limitation in our study. Although we conducted thorough diagnostic assessments, there is a possibility of misclassifying certain PFO cases, which could impact the broader applicability of our results. This limitation underscores the need for caution when interpreting our findings and highlights the importance of further research in this area. Fourth, for patients with a history of DOAC use who presented with acute large vessel occlusion, we did not analyze the differences in DOAC concentrations between the two groups. Laboratory monitoring of coagulation parameters alone cannot accurately assess the anticoagulant activity and bleeding risk associated with DOACs, potentially introducing bias into the occurrence of bleeding complications. Fifth, patients in the RESCUE BT trial were not randomized based on stroke subtype, such as cardio-embolic versus other etiologies. Determining the exact etiology of LVO solely based on non-invasive imaging before endovascular therapy can be challenging. Consequently, there might have been heterogeneity in stroke subtypes within the cardio-embolism subgroup, which could have influenced the treatment response to tirofiban. Future studies incorporating more stringent diagnostic criteria and advanced imaging techniques could provide a more precise understanding of tirofiban's role in cardioembolic stroke. Lastly, it is crucial to acknowledge that this study primarily focused on a Chinese population, which exhibits a high prevalence of ICAD and may possess unique genetic, dietary, and vascular risk factor profiles. Therefore, the generalizability of our findings to other ethnic populations with different stroke etiologies and risk factors remains uncertain. Further research involving diverse populations is necessary to establish the external validity of our results.

## Conclusion

The secondary analysis conducted in the RESCUE BT trial, with a specific focus on the cardio-embolism subgroup, has provided concerning insights into the concomitant administration of tirofiban and EVT. Our analysis has revealed a significant association between tirofiban usage and an increased risk of sICH among patients with cardioembolic stroke. These findings emphasize the potential harm and heightened bleeding risk associated with tirofiban administration in this population. To confirm and further explore these observations, additional studies are warranted, aiming to validate these findings and investigate alternative strategies for optimizing outcomes in patients with cardioembolic stroke.

## Data Availability

All data relevant to the study are included in the article or supplement. Further inquiries on data availability can be directed to the corresponding authors.

## Abbreviations

RESCUE BT: Endovascular Treatment With versus Without Tirofiban for Patients with Large Vessel Occlusion Stroke
MCA: Middle cerebral artery
DOAC: Direct Oral Anticoagulant
mRS: Modified Rankin Scale
sICH: Symptomatic intracranial hemorrhage
EVT: Endovascular therapy
LVO: Large vessel occlusion
ASPECTS: Alberta Stroke Program Early CT Score
eTICI: Expanded Thrombolysis in Cerebral Ischemia
IVT: Intravenous thrombolysis
PFO: Patent Foramen Ovale
RoPe: Risk of Paradoxical Embolism
